# Genetic and pharmacological validation of TAK1 inhibition in macrophages as a therapeutic strategy to effectively inhibit TNF secretion

**DOI:** 10.1038/s41598-018-35189-7

**Published:** 2018-11-19

**Authors:** Scott A. Scarneo, Antoine Mansourati, Liesl S. Eibschutz, Juliane Totzke, Jose R. Roques, David Loiselle, David Carlson, Philip Hughes, Timothy A. J. Haystead

**Affiliations:** 1 Department of Pharmacology and Cancer Biology, Duke University School of Medicine, Durham, NC 27710 USA; 2 Clinical and Translational Science Institute, Duke University School of Medicine, Durham, NC 27710 USA; 3 Lineberger Comprehensive Cancer Center, University of North Carolina at Chapel Hill, Chapel Hill, NC 27599 USA

## Abstract

Immune challenge of invading macrophages at sites of infection is associated with release of TNF, which triggers a local cytokine storm as part of the normal inflammatory response. Whereas this response maybe beneficial in fighting off infections, similar responses triggered in autoimmune diseases contribute significantly to the underlying damaging pathology associated with these diseases. Here we show that Takinib, a highly discriminatory inhibitor of transforming growth factor Beta- activated kinase 1 (TAK1), selectively and potently reduces TNF production in pro-inflammatory THP-1 macrophages. A complete survey of 110 cytokines, showed robust loss of proinflammatory cytokine responsiveness to lipopolysaccharide (LPS) and interferon gamma (IFNγ) challenge in response to Takinib. The mechanisms of action of Takinib was recapitulated in TAK1 KO macrophages. TAK1 KO cells showed significant loss of TNF production as well as release of IL-6 in response to LPS challenge. Furthermore, Takinib blocked the ability of exogenously added LPS to promote phosphorylation of, c-Jun, p38 protein kinases as well as downstream transcription factors regulated by nuclear factor κ-light-chain-enhancer of activated B cells (NFκB). In a mouse LPS challenge model, Takinib significantly reduced TNF serum levels. Our findings demonstrate that Takinib has utility in the treatment inflammatory disease by locally suppressing TNF production from invading macrophages.

## Introduction

Tumor necrosis factor alpha (TNF) is a potent proinflammatory cytokine and is an important therapeutic target for several chronic inflammatory diseases such as Rheumatoid Arthritis (RA)^1^. Biologic based anti-TNF therapies such as Etanercept and Adalimumab have shown to dramatically and effectively reduce disease associated pathology by directly targeting of TNF itself in responsive patient populations^2–4^. However, these treatment modalities fail to treat all RA cases and significant side effects associated with anti-body mediated therapies have limited broader application of these ground-breaking therapies^5,6^. To date, relatively few small molecule drugs directly targeting TNF secretion have been discovered^7^.

Autoimmune disease such as RA are characterized by a hyperactivation of immune cells in the joints of patients resulting in elevated pro-inflammatory cytokines. Macrophages are critical mediators of the innate immune response and have been identified as a key cell type involved in antigen presentation and cytokine release^8–10^. Toll like receptors on macrophages respond only upon stimulation by extracellular factors such as pathogen associated molecular patterns (PAMPs) or damage associated molecular patterns (DAMPs)^11,12^. Response to PAMPS is often mediated by pro-inflammatory molecular pathways leading to release of pro-inflammatory factors, which in turn further activate innate immune cells and the adaptive immune response^13^. In diseases such as Rheumatoid Arthritis, the process normally used to fight pathogens can become dysregulated, engendering a hyper activated pro-inflammatory state^14,15^. This dysregulation often leads to heightened levels of pro-inflammatory cytokines in the joints of patients.

Here we investigate the protein kinase TAK1 (transforming growth factor Beta- activated kinase 1) and the role it plays in mediating the pro-inflammatory response of macrophages. TAK1 mediates pro inflammatory signal transduction, and is down stream of the TNF receptor I and TLR4 receptor signaling cascades^16,17^. Following receptor ligand binding TAK1 undergoes ubiquitination and phosphorylation with the aid of its binding proteins TAB1, 2, 3^18^. TAK1 activation then leads to phosphorylation of p38 and JNK, as well as activation NFkB promoting pro inflammatory gene transcription, cell survival and proliferation^19–21^. Previous studies in cancer cells show TAK1 inhibitors reduced phosphorylation of many downstream signaling molecules including p-IKK p-p-38, and p-c-Jun following TNF stimulation. Takinib, a novel TAK1 inhibitor, has been shown to potently inhibit TAK1 (IC50 of~9 nM) *in vitro* kinase assays and, unlike many other TAK1 kinase inhibitors, Takinib has an exquisite selectivity towards TAK1 over all other protein kinases in the human kinome^22^. In this study we show Takinib potently reduces pro-inflammatory phenotypes and functional responses of the TLR4 receptor in response to LPS challenge. This drug effect was also recapitulated in THP-1 cells following TAK1 knock out using CRISPR/CAS9 targeted deletion. Additionally, Takinib reduces TNF serum levels following LPS challenge in a murine model of sepsis.

## Results

### TAK1 inhibition by Takinib reduces the pro-inflammatory cytokine milieu following LPS+ IFNγ stimulation

**Figure 1:**
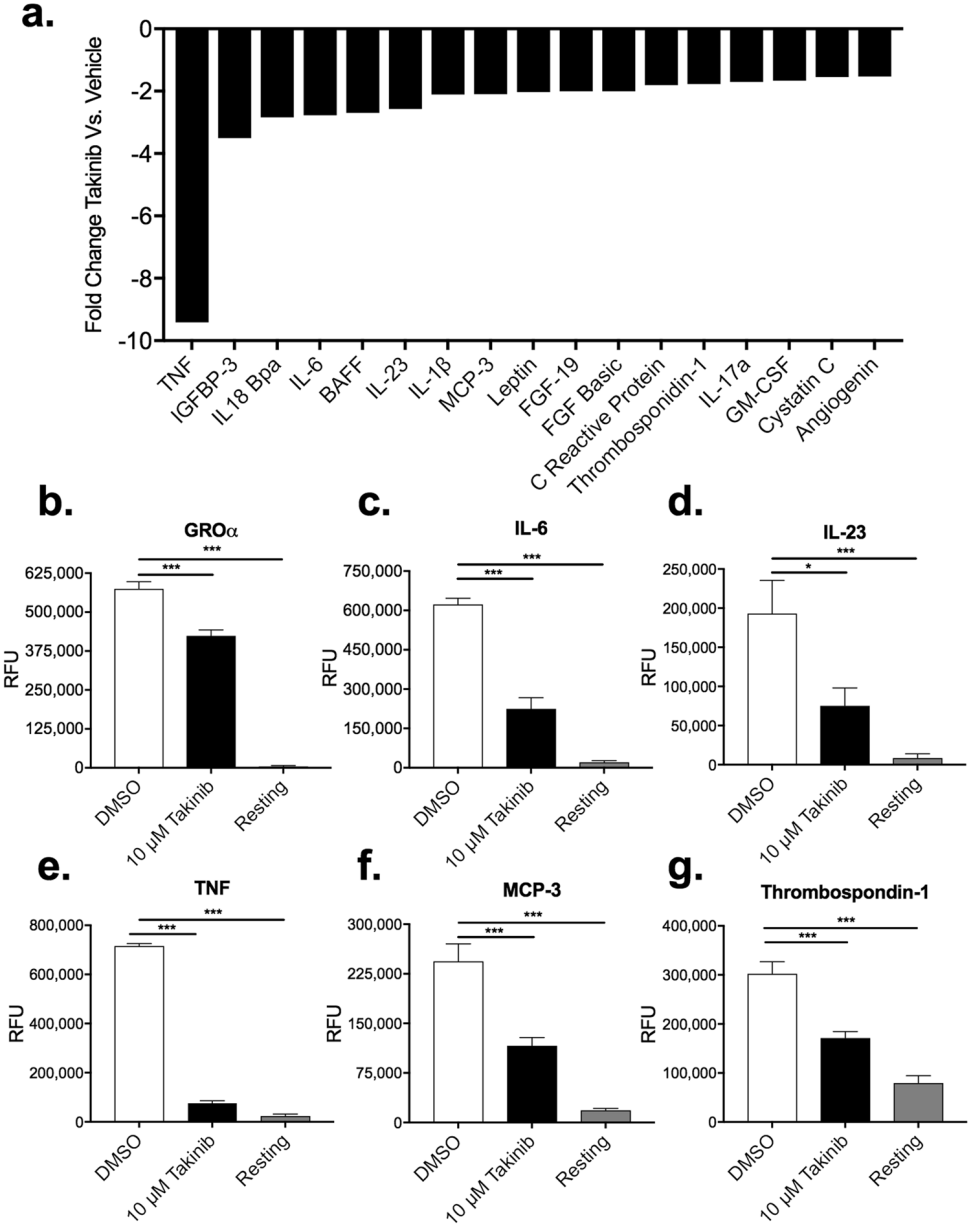
Takinib reduces the cytokine and chemokine response in pro inflammatory stimulated THP-1 human macrophages. THP-1 cells were differentiated in 100 nM PMA for 72 hours followed by a 48-hour rest period, cells were either left unstimulated (resting) or activated with LPS (10 ng/mL) and IFNγ (50 ng/mL) and treated with 10 μM Takinib or DMSO. 110 cytokine and chemokine proteins were profiled. Takinib reduces the expression of 17 different cytokines and chemokines in response to LPS and IFNγ stimulation (**a**). GROα, IL-6, IL-23, TNF, MCP-3 and Thrombospondin-1 levels in pro-inflammatory treated DMSO (n = 3 ± SEM), 10 μM Takinib (n = 4 ± SEM), or resting cells (n = 4 ± SEM) (**b–g**). (ANOVA with Dunnett’s) RFU = Relative Fluorescent Units *p < 0.05, **p < 0.01, ***p < 0.001.

TAK1 has been shown to mediate pro inflammatory signaling in immune cells^23,24^. We hypothesized that inhibition of TAK1 with Takinib would therefore significantly reduce pro-inflammatory cytokine secretion in stimulated macrophages. To test this hypothesis, we treated the human macrophage cell line THP-1, with Takinib in the presence of LPS and IFNγ. THP-1 cells were differentiated with 100 nM PMA for 72 hours, followed by a 48-hour rest period in PMA free media prior to either unstimulated (resting) or pro-inflammatory differentiation with LPS (10 ng/mL) and IFNγ (50 ng/mL). In an initial screen of 110 cytokines and chemokines, TNF secretion was reduced 9-fold in Takinib treated cells over control treated cells. Additionally, 17 other cytokines involved in the cytokine/chemokine response were reduced by 2-fold or greater when treated with Takinib (Fig. 1a). When compared to vehicle treated the following cytokines/chemokines were significantly reduced by Takinib treatment including GROα (P < 0.0004), IL-6 (P < 0.0001), IL-23 (P < 0.019), MCP-3 (P < 0.0006), TNF (P < 0.0001), and Thrombosbondin-1 (P < 0.0014) (Fig. 1b–g) and BAFF (P < 0.02), FGF-19 (P < 0.01), IGFBP-3 (P < 0.009), IL18Bpa (P < 0.008), MIP-3B (P < 0.001), Pentraxin 3 (P < 0.02) (Supplemental Fig. 1a). Additionally, the following proteins were significantly elevated in Takinib treated cells, Endoglin (P < 0.001), IL-33 (P < 0.008), IL-34 (P < 0.03), IP-10 (CXCL10) (P < 0.0019), MIG (CXCL9) (P < 0.03), PDGF-AA (P < 0.0001) and RBP-4 (P < 0.02) (Supplemental Fig. 1a). These findings are consistent with the role of TAK1 as a primary mediator of pro-inflammatory signaling.

### Takinib reduces TNF and IL-6 cytokine secretion in a dose dependent manner

**Figure 2:**
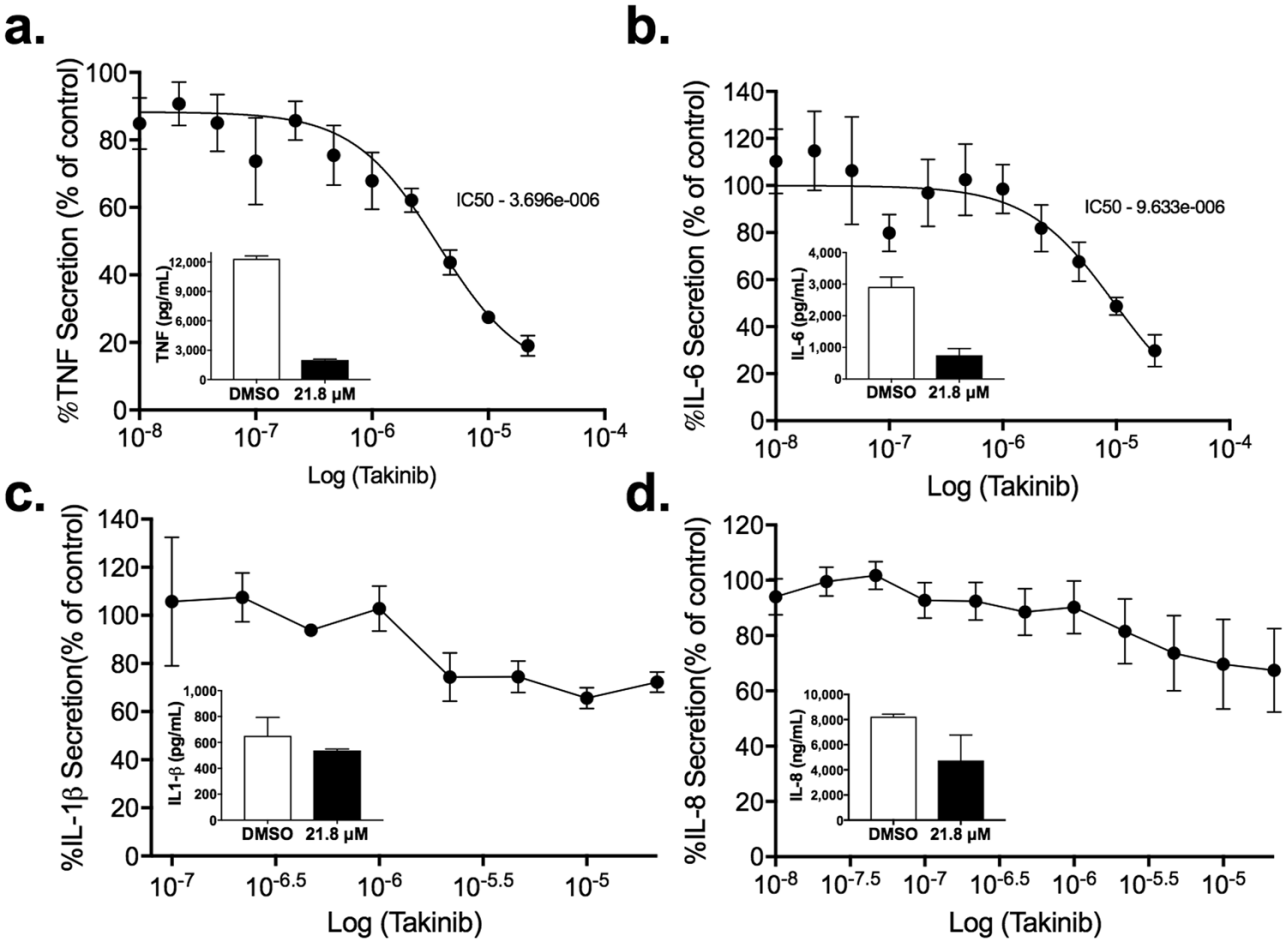
TAK1 inhibition reduces TNF, IL-6, IL-1β and IL-8 secretion in a dose dependent manner. THP-1 human macrophage cell line was differentiated in 100 nM PMA, followed by a 48-hour rest period. Following the rest period, cells were pro-inflammatory stimulated with LPS (10 ng/mL) and IFNγ (50 ng/mL) and treated with Takinib at varying doses or DMSO control. Takinib dose dependent effects of TNF (**a**), IL-6 (**b**), IL-1β (**c**), and IL-8 (**d**) secretion reported as a percent of vehicle control in THP-1 cells, (n = 3± SEM). Concentration of cytokines from DMSO treated or 21.8 μM Takinib are shown in the respective panel insets.

To investigate the cytotoxic effects of Takinib we stimulated THP-1 cells with LPS (10 ng/mL) and IFNγ (50 ng/mL) and treated the cells with Takinib at varying concentrations for 24 hours. Takinib was found to exert no cytotoxicity on pro-inflammatory stimulated THP-1 or U937 treated cells (Supplemental Fig. 2a). At 10 μM Takinib significantly reduces the secretion of TNF and IL-6 in LPS and IFNγ treated macrophages compared to control treated cells (Supplemental Fig. 2b,c). TNF and IL-6 levels were reduced in a dose dependent manner with Takinib with an IC50 of 3.66 and 9.6 µM respectively (Fig. 2b,c) Additionally, modest changes in IL-8 and IL-1β secretion were observed (Fig. 2e,f). This low cellular μM inhibition is consistent with the published 9 nM IC50 of Takinib against purified TAK 1 in standard kinase assays^22^. Takinib is an ATP competitive inhibitor and binds in the ATP binding pocket of TAK1, preventing autophosphorylation and further activation of the kinase^22^. However, in cells Takinib competes against intracellular ATP levels which can reach 10 mM^25^. At this intracellular ATP concentration, the predicted IC50 of Takinib in cell systems would correlate to ~2 μM, consistent with our cellular assays.

### Takinib inhibits the functional response of macrophages to pro-inflammatory stimuli

**Figure 3:**
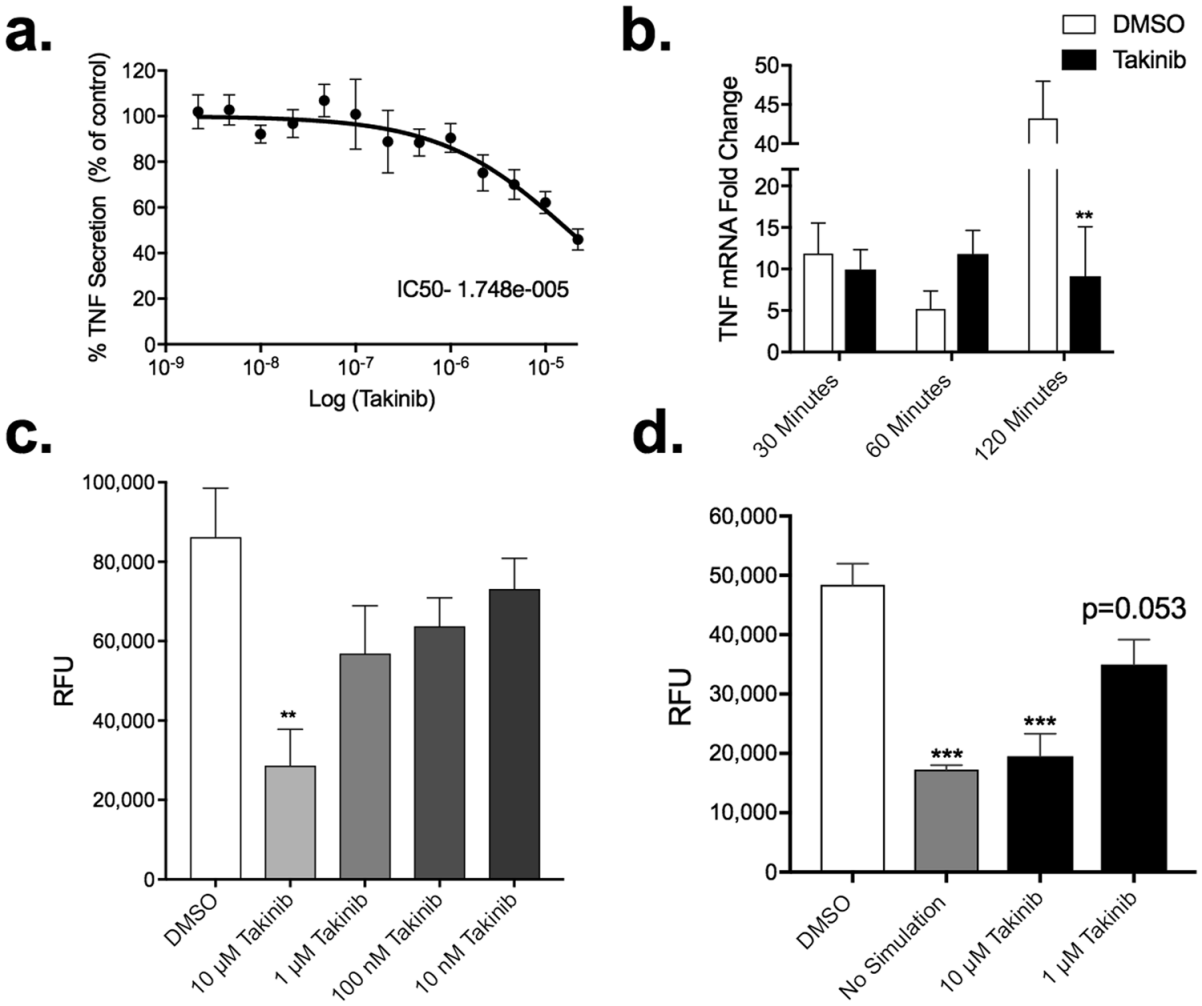
Takinib reduces the functional response of macrophages upon pro-inflammatory activation with LPS and IFNγ. Raw264.7 cells were plated at (0.25 × 10^6^/well) in 24-well plates and activated with LPS (10 ng/mL) and IFNγ (50 ng/mL) and treated with the indicated Takinib concentration or vehicle control for 24 hours (**a**). RAW 264.7 cell were plated at (3 × 10^6^/plate) and serum starved for 4 hours. Cells were pretreated with either 10 μM Takinib or vehicle for 30 minutes followed by LPS (10 ng/mL) stimulation for the indicated times. mRNA levels compared to vehicle (n = 4± SEM) (**b**). Raw264.7 cells were plated at (0.75 × 10^6^/well) in a 96-well Boyden migration chamber in serum free media. Cells migrated towards, 10% fetal bovine serum, media chambers for 18 hours and total cell migration determined (**c**). (One way ANOVA with Dunnett’s, n = 3± SEM). Reduced NO production following LPS + IFNγ stimulation and treatment with Takinib in RAW 264.7 murine macrophage cells (One-way ANOVA with Dunnett’s, n = 3± SEM) (**d**). RFU = Relative Fluorescent Units *p < 0.05, **p < 0.01, ***p < 0.001.

To confirm Takinib also reduced TNF levels in murine macrophage cell line, we tested the dose response of RAW 264.7 murine macrophages against varying doses of Takinib. Proinflammatory stimulated cells showed a reduction in TNF secretion compared to vehicle treated cells (Fig. 3a). Following LPS treatment (10 ng/mL) TNF mRNA levels were significantly reduced 120 minutes post treatment in 10 μM Takinib treated cells compared to vehicle (P < 0.008) (Fig. 3b). Functional macrophage responses such as chemotaxis and nitric oxide production were evaluated in response to pro inflammatory stimuli and Taknib treatment. Migration plays a significant role in activated macrophages. Using a Boyden chamber, cells were serum starved followed by treatment with Takinib at the indicated concentration and relative migration followed over 18 hours. 10 μM Takinib greatly reduced migration of activated macrophages compared with vehicle control (P < 0.005) (Fig. 3c). Up regulation of reactive oxygen species (ROS) production is a hallmark of macrophage activation in response to pro-inflammatory stimuli. Specifically, inducible nitric oxide synthase (iNOS) has been shown to increase nitric oxide (NO) levels in activated macrophages^26^. In Takinib treated RAW 264.7 cells activated with LPS (10 ng/mL) and IFNγ (50 ng/mL), NO was significantly reduced at 10 μM compared to DMSO (P < 0.02) (Fig. 3d).

### Takinib reduces downstream p-38, c-Jun and NFκB phosphorylation following LPS stimulation

**Figure 4:**
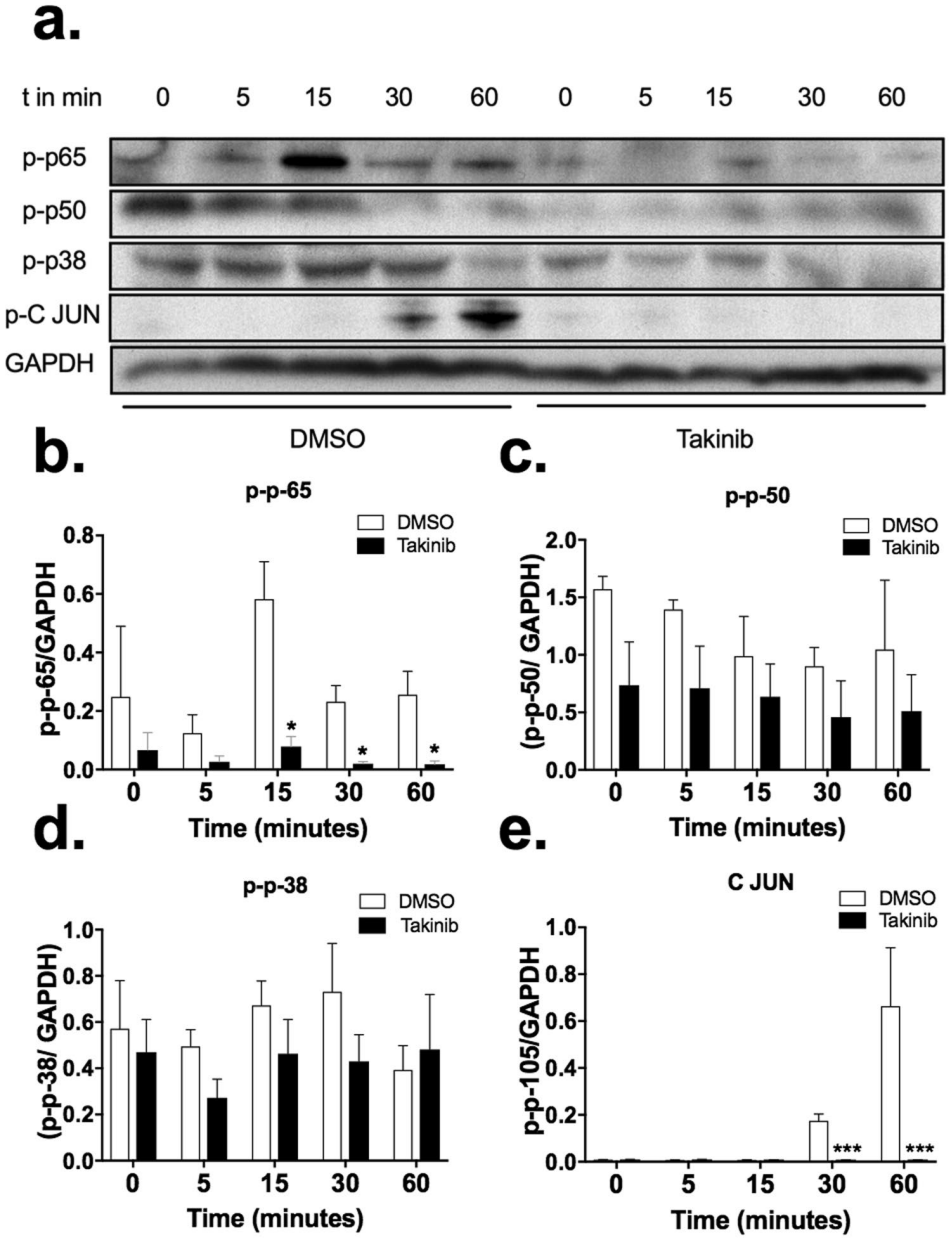
Takinib reduces phosphorylation of downstream p-38, c-Jun and NFκB in LPS stimulated Raw264.7 macrophages. Raw264.7 cells plated at (3 × 10^6^/plate) and serum starved for 4 hours. Cells were pre-treated for 30 minutes with either 10 μM Takinib or DMSO followed by LPS (10 ng/mL) stimulation for the indicated times. Representative images of downstream phosphorylation of p-65, p-50, p-38 and p-c-Jun were detected via western blot and normalized to GAPDH (**a**). Images represent one biological replicate of 3, from the same biological blot stripped and re probed for the indicated proteins. Quantitative data shown as (**b–e**) (Student t-test, n = 3± SEM). *p < 0.05, **p < 0.01, ***p < 0.001.

TAK1 has been shown to be downstream of numerous cytokines signaling cascades^27,28^. Here we show TAK1 mediates pro inflammatory signals from LPS-TLR4 stimulated RAW 264.7 murine macrophages through c-Jun, p-38, p50 and p65. Cells treated with TAK1 inhibitor, Takinib, showed reduced phosphorylation of downstream pro-inflammatory kinases (Fig. 4a,b–e). Significant reduction of phosphorylation of p65 at 15 (P < 0.018), 30 (P < 0.018) and 60 minutes’ (P < 0.042) post LPS stimulation was observed (Fig. 4a,b). Modest decreases in p-p50 and p-p38 phosphorylation was observed at 5 and 15 minutes (Fig. 4a,c,d). Additionally, phosphorylation of c-Jun was significantly reduced compared to Takinib treated macrophages, 30 (P < 0.0001) and 60 (P < 0.0001) minutes following LPS stimulation (Fig. 4a,e).

### TAK1 genetic knockout (KO) recapitulate Taknib’s therapeutic effect

**Figure 5:**
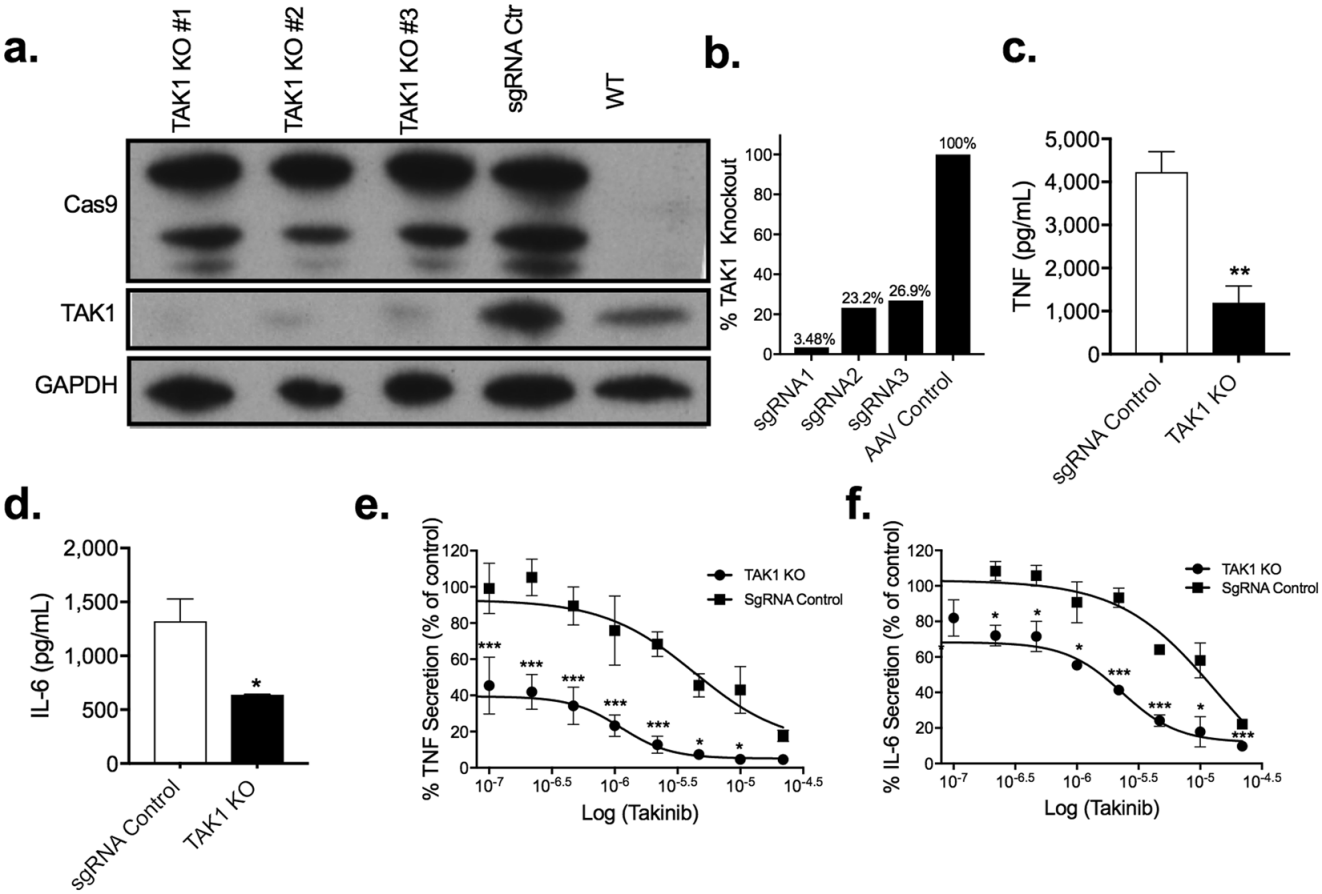
Genetic KO of TAK1 in THP-1 cells recapitulate Takinib’s inhibition following stimulation. Immuno-blot representing relative TAK1, Cas9 and GAPDH protein expression in TAK1 KO, sgRNA control THP-1 cells, and wild type THP-1 cells (**a,b**). Western blot represents one biological replicate from the same gel. Following THP-1 cell differentiation as previously described, LPS (10 ng/mL) and IFNγ (50 ng/mL) treated THP-1 TAK1 KO cells release significantly less TNF and IL-6 than sgRNA control cells (**c,d**) (Students t-test, n = 3± SEM). TAK1 KO and sgRNA control THP-1 cells were differentiated as previously described and pro-inflammatory activated, simultaneously cells were treated in a dose dependent manner with Takinib. TNF and IL-6 secretion reported as percent of vehicle control treated cells (**e**,**f**). (2 way ANOVA with Sidak’s, n = 3± SEM). *p < 0.05, **p < 0.01, ***p < 0.001.

To test the hypothesis that TAK1 mediates pro-inflammatory signal transduction and subsequent transcriptional events we created a TAK1 KO THP-1 cell line. Using the CRISPR/CAS9 system TAK1 expression was knocked out in THP-1 macrophages. Three target sgRNA’s were experimentally evaluated for their ability to knockout TAK1 expression. sgRNA 1 successfully abolished ~97% of TAK1 expression compared to CRISPR/CAS9 control as determined by Western blot without significant effect on over all cellular viability (Fig. 5a,b). To control for any cellular changes introduced by the CRISPR/CAS9 system, cells were transfected with CRISPR/CAS9 and random sgRNA. Deletion of TAK1 significantly reduced TNF (P < 0.007) and IL-6 (P < 0.03) secretion in LPS and IFNγ stimulated cells by 75% and 50% respectively (Fig. 5c,d). Furthermore, TAK1 KO THP-1 cells pro-inflammatory stimulated followed by varying concentrations of Takinib, also significantly reduced TNF and IL-6 secretion compared to sgRNA control cells (Fig. 5e,f). Given the degree of selectivity of Takinib towards TAK1 over all other protein kinases, we attribute the mild dose dependent response due to the incomplete knock down of TAK1 in the THP-1 cells.

### Takinib is well tolerated in mice and therapeutically active in primary bone marrow derived macrophages

**Figure 6:**
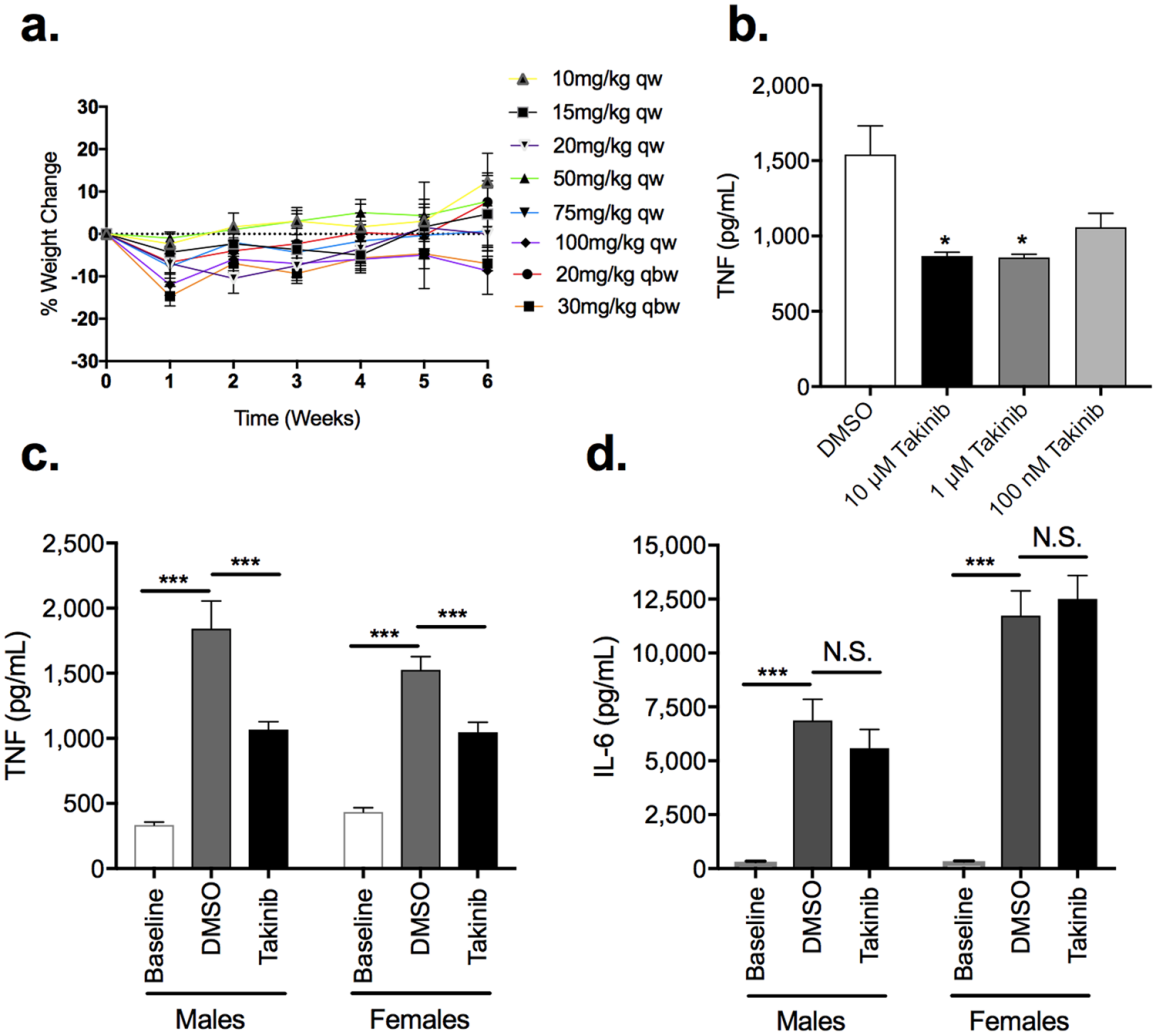
Takinib reduces TNF in a murine LPS challenge. FVB mice treated i.p. with varying concentrations of Takinib, once (qw) or twice weekly (qbw), caused no significant adverse events or weight change over 8 weeks of chronic treatment (**a**). Bone marrow derived macrophages (BMDM’s) were plated at (2 × 10^6^/well) in a 24-well plate and treated with M-CSF (20 ng/mL) for 72 hours followed by a 24 hour rest period prior to activation with LPS (10 ng/mL) and IFNγ (50 ng/mL) and treatment with Takinib, at the indicated concentrations, or vehicle for 24 hours. Cytokine production was sampled from cell supernatant and examined by ELISA (**b**) (One-way ANOVA with Dunnett’s, n = 3± SEM). C57/bl6 male and female mice were sacrificed prior to LPS challenge and baseline TNF and IL-6 serum concentrations were examined by ELISA (**c,d**) (Student t-test, n = 3± SEM, 3 males, 3 females). C57/bl6 male and female mice were injected intraperitoneally (i.p.) with LPS (100 μg/kg in PBS) followed immediately by vehicle or Takinib (50 mg/kg in DMSO). 1 hour later, mice were sacrificed and blood samples clotted in centrifuge for 10 minutes at 3,000 rpm. Concentration of serum cytokines TNF and IL-6 determined by ELISA (**c,d**) (Student t-test, n = 5± SEM).

The maximum tolerated dose (MTD) of Takinib was investigated to determine the effects of chronic TAK1 inhibition. Here, mice were treated with Takinib either once or twice weekly for 8 weeks at varying concentrations of Takinib. No significant weight change was observed in mice treated with Takinib (Fig. 6a). Additionally, primary bone marrow derived macrophages isolated from bone marrow of C57/bl6 mice were stimulated with LPS (10 ng/mL) and IFNγ (50 ng/mL). Treatment with 10 μM and 1 μM Takinib showed significant reduced TNF cytokine levels compared with vehicle treated (P < 0.03, P < 0.02 respectively) (Fig. 6b).

### Takinib reduces TNF cytokine serum levels in a murine sepsis model

In a murine sepsis model, mice were challenged with i.p. injection of LPS (100 μg/kg) and treated with either Takinib (50 mg/kg) or DMSO for 1-hour post LPS injection. Slightly higher baseline levels of TNF were observed in female mice over male mice at baseline (P < 0.07) (Fig. 6c,d). To insure a robust cytokine increase is produced by the LPS challenge model we tested TNF and IL-6 cytokine serum levels from baseline to 1-hour post LPS challenge. A ~4- and 45-fold increase in TNF and IL-6 cytokine levels were observed 1-hour post LPS challenge compared to baseline. These results indicate a significant response to LPS was observed (Fig. 6c,d). In Takinib treated mice following LPS challenge a significant reduction of TNF compared to vehicle treated mice was observed in males (P < 0.008) and females (P < 0.005) (Fig. 6c). However, Takinib did not reduce IL-6 serum cytokine levels in males or females compared to vehicle treated (Fig. 6d). Serum levels of Takinib at 1-hour post LPS challenge was quantified and reported (Supplemental Fig. 3a).

## Discussion

Our studies support the hypothesis that TAK1 may be a fruitful drug target in TNF mediated diseases. We showed that inhibition of TAK1 in macrophages by Takinib potently and dramatically reduces TNF secretion as well as numerous other pro inflammatory cytokines and chemokines. Furthermore, deletion of TAK1 in the THP-1 cells completely recapitulates the effects of Takinib in terms of responses to LPS + IFNγ. Similarly, Takinib treated cells remain viable indicating TAK1 can be chronically targeted *in vivo*. Furthermore, Takinib significantly reduces TNF cytokine serum levels in LPS challenged mice indicating its potential use as an anti-TNF small molecule therapy.

Response to pro-inflammatory stimuli is a necessary biological response to pathogens. However, in diseases such as RA, this process becomes chronic leading to extended hyper activation of pro-inflammatory immune cells. Complete inhibition of the immune system poses potentially lethal side effects, as patients may become susceptible to pathogens without a robust immune response. Our findings demonstrating that TAK-1 inhibition, either pharmacologically or by genetic deletion, greatly reduces TNF secretion from immune cells as well as other pro-inflammatory cytokines without complete loss of all pro-inflammatory cytokine expression, strongly suggest that drugs targeting TAK-1 such as Takinib, can be used as an effective anti-TNF therapy, while leaving a functional immune system intact. Targeting TAK1 as a therapeutic strategy is likely to have several advantages over existing biological based approaches or other kinase inhibitor-based strategies targeting down-stream elements in the TNF mediated signaling. In the case of biologics such as Etanercept and Adalimumab, these highly effective drugs remove circulating TNF, and in responsive patients this is sufficient to alleviate the symptoms of chronic rheumatoid arthritis^29–31^. However, therapy is limited to a few years and once a patient has been exposed to these biologicals, they cannot be used again due to immune sensitization^32–34^. Patients are then forced to use older more problematic drugs such as methotrexate or chloroquine^35^.

Alternate kinase based anti-inflammatory strategies have yet to match the success and efficacy achieved with anti-TNF biologicals. Inhibitors targeting kinases down stream of TAK1 such as p38mapk and NFκB, despite extensive efforts have yet to make progress beyond early stage clinical trials^36–39^. Lack of target specificity and unexpected side effects have often been attributed to the failure of seemingly promising compounds^39^. At the time of writing the most promising kinase inhibitors as alternatives to the biologicals are the JAK/STAT inhibitors such as tofacitinib^40,41^. Several second generation JAK inhibitors have also received FDA approval or are in late stage clinical development^42,43^. Several factors have limited the use of JAK inhibitors and their side effect profiles are somewhat different than their biological counterparts Etanercept and Adalimumab^44–46^. Because of this, approved JAK-STAT inhibitors are indicated for RA patients who cannot tolerate or are resistant to methotrexate^47^. Significant differences seen in the side effect profiles for patients treated with JAK/STAT inhibitors are increased risk of infections such as Herpes, cellulitis, pneumonia and urinary tract infection as well as increased risk for tuberculosis^48^. Based on these clinical findings, and the extensive clinical data derived from the almost two decades of experience with anti-TNF biologics, we and others hypothesize that selectively inhibiting TNF mediated signaling, may be a viable alternative to the JAK/STAT inhibitors. Selective inhibitors of TAK1 could hold great promise as an alternative to the current anti-TNF biologicals, since compounds like Takinib are non-biological small molecules that can be readily developed into oral formulations. Additionally, genetic knockout of TAK1 suggests that the actions of drugs selectively targeting the protein kinase are likely to be confined to TNF signaling, and at worst mimic those already understood with biological antibody-based therapies.

## Materials and Methods

### Animal Care

Male and female C57/bl6 mice were bred in-house or purchased from The Jackson laboratory (Bar Harbor, ME, USA). All experiments were approved and carried out in accordance of the Duke University and University of North Carolina- Chapel Hill, Institution Animal Care and Use Committee (IACUC) and conformed to the National Institutes of Health Guide for the Care and Use of Laboratory Animals. Mice were housed in a temperature and humidity-controlled facility under 12-hour light/dark cycle (lights on at 7 am) and access to food and water *ad libitum*.

### Lipopolysaccharide Challenge

C57/bl6 male and female mice were injected with LPS (100 μg/kg) i.p. in combination with Takinib (50 mg/kg) or vehicle. Mice were survived 60 minutes’ post LPS challenge. Blood was collected via cardiac puncture and serum isolated and stored at −80 °C until analysis. 5 mice per experimental condition per sex.

### Maximum Tolerated Dose (MTD)

FVB/NJ mice were injected at 10,15, 20, 50, 75 and 100 mg/kg of Takinib weekly (qw) and 20 and 30 mg/kg twice weekly (qbw) for 8 weeks. Weight change was recorded for each animal over the course of treatment. 3 mice were used per experimental condition.

### Bone Marrow Derived Macrophages

Acutely cultured bone marrow derived macrophages were obtained from tibia and femora of naïve male C57Bl/6J mice. Immature bone marrow cells were culture for 72 hours in macrophage colony-stimulating factor (M-CSF) (20 ng/mL) supplemented culture media to induce macrophage differentiation. Following 72-hour rest period in media cells were treated with Takinib and LPS as described below.

### Pharmacokinetics

All samples were frozen and stored at −80C. Before quantifying the Takinib in serum, a standard curve was made using HS-219, a close structural analog of Takinib that serves as an internal standard. This internal standard solution was used for tissue homogenization. LCMS analysis was performed at the Duke Proteomics and Metabolomics Core Facility analogous to^49^. The final concentration of Takinib in the plasma was calculated per ml of plasma.

### RNA purification, cDNA synthesis, and real-time quantitative PCR

Total RNA extraction was performed using Total RNA Isolation kit (Qiagen) according to manufacturer’s instructions. cDNA was synthesized using the Bio-Rad iScript^TM^ cDNA Synthesis Kit and stored at −20 °C until further analysis. All results were normalized to GAPDH mRNA levels. Primers for TNF F 5′-GCCTCTTCTCATTCCTGCTTG-3′, R 5′-CTGATGAGAGGGAGGCCATT-3′ and housekeeping gene GAPDH F 5′-GTTGTCTCCTGCGACTTCA-3′ and R 5′GGTGGTCCAGGGTTTCTTA-3′. mRNA levels were quantified on Bio-Rad CFX Connect using Bio-Rad iTaq Universal SYBR Green Supermix (Bio-Rad, Hercules, CA). Cycling conditions were as follows: 1 cycle at 95.0 °C for 2 minutes followed by 40 cycles of 95.0 °C for 5 seconds and annealing temperature for 30 seconds. Samples were run in triplicate and the average fold change was calculated using 2^−∆∆Cq^ method.

### Cell culture

THP-1 and RAW 264.7 cells were obtained from American Type Culture Collection. Cells were incubated at 37 °C in 5% CO_2_. THP-1 and U-937 cells were cultured in RPMI 1640X, 10% FBS, 1% Penicillin-Streptomycin (PS), HEPES, Pyruvate, Glucose and BME. RAW 264.7 were cultured in DMEM medium containing 10% FBS and 1% PS.

### Macrophage Differentiation

THP-1 cells were treated with 100 nM phorbol 12-myristate 13-acetate (PMA) for 72 hours in RPMI 1640X media. Cells were rested in PMA free media 48 hours prior to treatments. LPS (10 ng/mL) and IFNγ (50 ng/mL) were used for pro-inflammatory stimulation. IL-4 (10 ng/mL) and TGF-β (10 ng/mL) for an anti-inflammatory induced state.

### Cytokine/ Chemokine proteome Profile

THP-1 Cells were differentiated as previously described in this manuscript. Following differentiation, cells were treated with 10 μM Takinib or DMSO. 24 hours after treatment, supernatant was added to Human Cytokine XL proteome array (R&D Systems). Cytokine kit was conducted in accordance with manufacturer protocol. Chemiluminescence was used to visualize protein quantities.

### TAK1 Gene Editing

THP-1 cells were infected with CRISPR/CAS9 constructs with a blasticidin resistance gene. Cells were treated with blasticidin for 5 days following transfection to select transfected cells. Following stable cas-9 expression cells were transfected with lentiguide- puromycin sgRNA targeting TAK1 gene sequence. The following sgRNA sequences were used.

sgRNA 1–5′ CTCACCGGCCGAAGACGAGG 3′

sgRNA 2–5′ CGACTACAAGGAGATCGAGG 3′

sgRNA 3–5′ CATCTCACCGGCCGAAGACG 3′

### Western Blot Analysis

Cells were lysed (50 mM Tris, 150 mM NaCl, 1 mM EDTA, 1%Triton-X100, 1 mM DTT, complete protease and PhosStop phosphatase inhibitor) after indicated treatment and run on Criterion XT Tris-HCl gel 4–15% gradient (Bio-Rad). Following transfer to PVDF membrane and blocking in 5% non-fat dry milk in TBST, membranes were incubated with antibody overnight. After incubation with secondary antibody, chemiluminescence was used to visualize bands. Exposure times for p-65, and p-50 (60 seconds), p-38 and p-c-Jun (180 seconds), Cas9(30 seconds), TAK1 (120 seconds) and GAPDH (15 seconds).

### ELISA cytokine assays

THP-1 cells were plated and differentiated as previously described. Cells were stimulated with LPS and IFNγ in the presence of Takinib or DMSO. 24 h after treatment, supernatant was added to ELISA plates (IL-6, IL-8, TNF, IL-1B, Ready- SET-Go! from Thermo). ELISA was conducted in accordance with the manufacturer’s protocol. Absorbance was measured at 450 and 570 nm. Experiments were repeated 3 times.

### Migration Assay

RAW 264.7 cells were serum starved for 6 hours prior to treatment. ~50,000 cells were seeded in serum free media at the indicated concentrations of Takinib. 10% FBS was used as a chemo attractant in the lower chambers. Cells migrated for 18 hours through a 5 μm Boyden Chamber (Sigma). Cell migration was measured by CellTiter Glow 2.0 assay (Promega).

### Nitric Oxide Assay

RAW 264.7 cells were plated and treated with Takinib at varying concentrations. Following treatment cells were activated with 10 ng/mL of LPS and 50 ng/mL of IFNγ. 24 hours post activation; nitric oxide production was measured utilizing Nitric Oxide Synthase Detection System kit (Sigma) according to the manufacturer’s protocol. Absorbance was measured at 515 nm.

### Cell Viability Assay

Cells were cultured and treated as previously described. Cell death was quantified using Cell Titer Glo 2.0 (Promega) according to the manufacturer’s protocol.

### Quantification and Statistical Analysis

Graphpad Prism 7 was used for statistical analysis of viability, ELISA and Cytokine XL proteome assays, TAK1 KO analysis, LPs challenge. For each analysis, total n and SEM are presented in the figure legend. Curves were plotted using variable slope (four parameters) non-linear fit. An alpha of 0.05 was used for all statistical analysis.

## Electronic supplementary material

Supplemental Figure 1

## Data Availability

The datasets generated during and/or analyzed during the current study are available from the corresponding author on reasonable request.
