# Supplementary material for: Genetic and pharmacological validation of TAK1 inhibition in macrophages as a therapeutic strategy to effectively inhibit TNF secretion
Source: Sci Rep. 2018 Nov 19;8:17058. doi: 10.1038/s41598-018-35189-7 (PMC6242965; doi:10.1038/s41598-018-35189-7)
Supplement: Supplementary file 1 — Supplemental Figure 1 [file 41598_2018_35189_MOESM1_ESM.pdf]

Genetic and pharmacological validation of TAK1 inhibition in macrophages as a therapeutic strategy to effectively inhibit TNF secretion.

Scott A. Scarneo[1], Antoine Mansourati[2], Liesl S. Eibschutz[1], Juliane Totzke[1], Jose R. Roques[3], David Loiselle[1], David Carlson[1], Philip Hughes[1], Timothy A.J. Haystead[1]

[1]Department of Pharmacology and Cancer Biology, Duke University School of Medicine, Durham, NC 27710

[2] Clinical and Translational Science Institute, Duke University School of Medicine, Durham, NC 27710

[3]Lineberger Comprehensive Cancer Center, University of North Carolina at Chapel Hill, Chapel Hill, NC 27599

Phone Number: (919) 613-8609

Fax Number: 919-613-8600

Email: Timothy.Haystead@Duke.edu

SFigure 1.

	Resting		Stimulation- DMSO Treated		Stimulated- Takinib (10uM) Treated	
	**Average**	**SD**	**Average**	**SD**	**Average**	**SD**
Adiponectin	40341	30643	46318	27316	41479	33667
Apolopoprotein A-1	72102	17017	88892	19271	77998	63735
Angiogenin	254531	29108	54275	5783	35515	31614
Angiopoietin-1	18079	13443	13727	1752	10518	10108
Angiopoietin-2	162552	14416	200217	2960	171486	124990
BAFF	39965	5689	85884	10633	31833	27037
BDNF	132602	22638	201710	25216	150752	112247
C5a	35092	7163	15211	3312	13203	16116
CD14	598655	24126	404056	34470	278610	193121
CD30	64822	26702	20423	19968	26746	25537
CD40 Ligand	55527	42648	86709	46921	76003	51146
Chitinase-3 like 1	1065251	19820	1003089	27587	1076677	51492
Compliment Factor D	811631	16394	703349	44829	688509	143079
C Reactive Protein	29482	9729	21602	7542	11988	13845
Cripto-1	24344	13635	7220	5177	11616	14999
Cystatin C	648505	67054	286068	113223	185517	154045
Dkk-1	115995	10365	92135	17388	78939	66516
DPPIV	27375	10573	10556	18157	23628	27623
EGF	46947	15092	9399	8432	16286	21703
Emmprin	367747	28523	538520	64329	439446	303258
ENA-78	15918	18242	12813	14785	13220	9769
Endoglin	155914	26535	279340	66669	562395	52544
Fas Ligand	29421	16796	22347	11705	15370	13761
FGF Basic	29603	13313	21563	2894	10771	8276
FGF-7	20464	10965	0	0	0	2963
FGF-19	374742	33842	396005	60958	197501	66831
Flt-3 Ligand	21835	6165	457	792	6605	7706
G-CSF	31510	11476	12031	17958	15092	18614
GDF-15	949257	16556	881790	50870	825833	126109
GM-CSF	75165	18875	30833	27545	18524	26179
GROα	4626	5721	574611	39510	423904	37736
Growth Hormone	8516	11082	3426	3022	7958	9237
HGF	35858	20755	48173	7647	65608	24047
ICAM-1	26926	10262	229691	30378	240939	43641

IFN-y	54571	21481	587554	8532	595438	30449
IGFBP-2	33401	8373	0	0	1684	2777
IGFBP-3	23607	12075	29650	1400	8466	8691
IL-1a	104886	16972	161969	22908	143308	84312
IL-1B	29397	9530	79484	23059	37800	27311
IL-1ra	300399	14082	576402	77587	486804	187256
IL-2	54603	15191	9130	8220	14979	17582
IL-3	48182	12162	4175	7231	13315	18751
IL-4	22960	11979	29397	3861	24701	16146
IL-5	15864	12895	34248	17175	42976	37376
IL_6	20674	12534	622779	41243	224701	85575
IL-8	329453	31717	982361	34692	1050060	32933
IL-10	16534	16599	9530	2598	8549	9732
IL-11	27929	15623	5115	7804	7290	9485
IL-12p70	19873	15478	1245	2156	8971	10747
IL-13	20337	11868	0	0	4727	4114
IL-15	27260	10063	0	0	8190	9533
IL-16	37586	14436	13189	14496	36027	24236
IL-17a	294527	28071	292947	142226	172006	31837
IL18 Bpa	347172	67769	392448	63293	138364	87221
IL-19	234	468	3543	3895	3905	6718
IL-22	55570	13898	43771	9856	36532	11162
IL-23	8519	10621	193063	73404	75073	46061
IL-24	20337	8727	52077	5846	66168	15546
IL-27	40430	12197	73231	13719	51401	20853
IL-31	17169	11940	0	0	8228	10371
IL-32	45588	16065	17269	22318	48292	42789
IL-33	20688	8220	3654	6329	27359	8042
IL-34	26443	14838	0	0	13035	7789
IP-10	57633	14924	1004996	7433	1071683	18055
I-TAC	44033	16335	1030441	18863	1053190	36183
Kallikrein 3	141548	16101	191691	33075	142650	88615
Leptin	76	151	14263	8766	7037	6091
LIF	2438	2817	4356	5470	9018	6547
Lipocalin-2	20877	6451	24158	8284	59540	26107
MCP-1	47542	8176	772457	23340	790308	51750
MCP-3	18309	6046	243966	45534	116182	25028
M-CSF	30946	13208	33053	23952	41806	19189
MIF	517949	42457	599693	70396	577895	101587

MIG	29910	11828	957167	40288	1013617	7978
MIP-1a/MIP-1B	29364	21224	839755	31259	810190	21094
MIP-3a	773073	28769	1012100	10403	1022951	15367
MIP-3B	55563	6674	830341	32501	619228	45713
MMP-9	846839	54364	766493	23327	762852	66893
Myeloperoxidase	5200	6152	14034	3819	25702	19021
Osteopontin	993569	25781	969567	47893	989587	16879
PDGF-AA	632941	15052	582007	13584	734452	20830
PDGF-AB/BB	6648	10365	2517	4359	9885	8464
Pentraxin 3	197469	17916	599730	65691	489501	20344
PF4	18076	12251	10664	17183	13399	3891
RAGE	19759	14087	4649	8051	18209	7959
RANTES	50061	22992	767312	37730	772329	24229
RBP-4	27157	10664	4792	8137	21212	5086
Relaxin-2	59797	8513	49217	20523	68946	20126
Resistin	72774	7091	30751	30325	58974	19330
SDF-1a	147587	39635	336746	14750	252570	88483
Serpin E1	69317	32710	115489	19977	87286	41862
SHBG	62353	27269	66680	26396	64044	30822
ST2	12626	8169	18717	12879	29492	12977
TARC	39270	12763	83520	26354	58478	19557
TFF-3	20001	11250	28140	25565	21454	1978
TfR	64183	16906	118820	63897	116027	46482
TGFα	12479	13739	0	4319	5088	3914
Thrombosponidin-1	79482	30272	302317	42378	171059	26701
TNF	23444	16569	715425	17592	75983	18729
uPAR	824197	17831	817217	25885	837024	22486
VEGF	63840	13520	288231	29465	242090	41967
Vitamin DBP	67549	23075	119833	22036	133162	60202
CD31	300755	14960	543964	54433	567060	180335
Tim-3	869682	15859	935936	55407	905344	27447
VCAM-1	53235	14046	746964	75171	703970	49763

SFigure 2

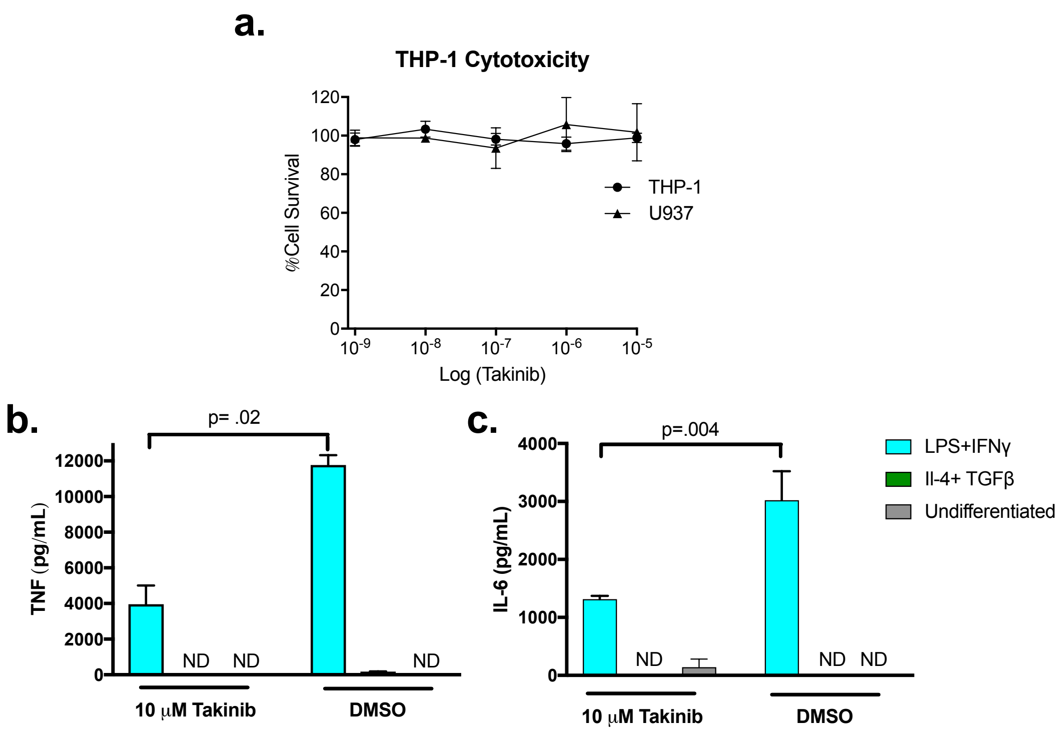

SFigure 3

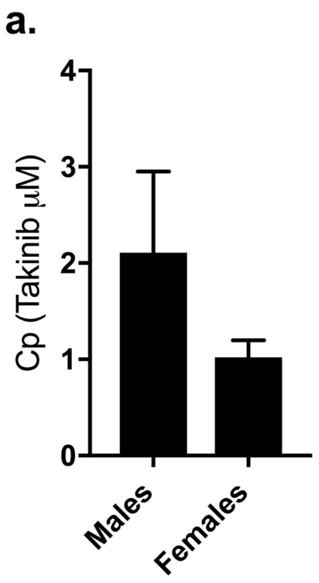

Supplemental Figures

SFigure 1.

Takinib alters the cytokine and chemokine response in pro inflammatory stimulated THP-1 human macrophages. 110 cytokine and chemokine proteins were profiled. Cytokine and chemokine response in Takinib treated, DMSO control and resting THP-1 cells (n=3 ± SD), 10μM Takinib (n=4 ± SD), or resting cells (n=4 ± SD).

SFigure 2.

Takinib exerts no cytotoxic effect on pro-inflammatory stimulated macrophages. THP-1 and U937 human macrophage cell lines were differentiated in 100 nM PMA, followed by a 48 hour rest period. Following the rest period, cells were pro-inflammatory stimulated with LPS (10 ng/mL) and IFNγ (50ng/mL). Cytotoxic effects of Takinib in pro-inflammatory macrophages **(a)** (n=3± SEM).10μM Takinib significantly reduces TNF and IL-6 secretion in THP-1 human macrophages. Whereas resting and anti-inflammatory (IL-4 +TGFβ) treated cells showed no significant up regulation of TNF and IL-6 cytokines nor any Takinib induced changes (**b,c**).

SFigure3

Plasma concentration of Takinib (Cp) of male and female mice treated in LPS challenge (**a**.)
